# Identification and Enhancement of HLA-A2.1-Restricted CTL Epitopes in a New Human Cancer Antigen-POTE

**DOI:** 10.1371/journal.pone.0064365

**Published:** 2013-06-04

**Authors:** Yi-Hsiang Huang, Masaki Terabe, C. David Pendleton, Deborah Stewart Khursigara, Tapan K. Bera, Ira Pastan, Jay A. Berzofsky

**Affiliations:** 1 Institute of Clinical Medicine, Infection and Immunity Research Center, National Yang-Ming University, Division of Gastroenterology, Taipei Veterans General Hospital, Taipei, Taiwan; 2 Vaccine Branch, Center for Cancer Research, National Cancer Institute, National Institutes of Health, Bethesda, Maryland, United States of America; 3 Laboratory of Molecular Biology, Center for Cancer Research, National Cancer Institute, National Institutes of Health, Bethesda, Maryland, United States of America; Dana-Farber Cancer Institute, United States of America

## Abstract

Identification of CD8^+^ T cell epitopes that can induce T cells to kill tumor cells is a fundamental step for development of a peptide cancer vaccine. POTE protein is a newly identified cancer antigen that was found to be expressed in a wide variety of human cancers, including prostate, colon, lung, breast, ovary and pancreas. Here, we determined HLA-A2.1-restricted cytotoxic T lymphocyte (CTL) epitopes in the POTE protein, and also designed enhanced epitopes by amino acid (AA) substitutions. Five 9-mer peptides were first selected and their binding affinity to HLA-A2 molecules was measured by the T2 binding assay. POTE 272–280 and POTE 323–331 showed the strongest HLA-A2 binding affinity. AA substituted peptides POTE 252-9V (with valine at position 9), POTE 553-1Y (with tyrosine at position 1) and POTE 323-3F (with phenylalanine at position 3) conferred higher affinity for HLA-A2, and induced CTL responses cross-reactive with wild type antigens. While POTE 252-9V was the strongest in this respect, POTE 323-3F had the greatest increase in immunogenicity compared to wild type. Importantly, two modified epitopes (POTE-553-1Y and POTE-323-3F) induced CTLs that killed NCI-H522, a POTE-expressing HLA-A2^+^ human non-small cell lung cancer cell line, indicating natural endogenous processing of these epitopes. In conclusion, the immunogenicity of POTE epitopes can be enhanced by peptide modification to induce T cells that kill human cancer cells. A combination of POTE 553-1Y and POTE 323-3F epitopes might be an attractive vaccine strategy for HLA-A2 cancer patients to overcome tolerance induced by tumors and prevent escape.

## Introduction

Most cancers cannot be curatively resected except when detected early. Other nonsurgical approaches, such as radiotherapy or chemotherapy, also affect normal cells and result in side effects that limit treatment. In addition, all treatments for recurrent or metastatic cancer are palliative. Consequently, development of novel systemic approaches to treat advanced, recurrent or metastatic cancer is needed. Immunotherapy may have great potential as a promising treatment for cancer patients because of its specificity and freedom from toxic effects of chemotherapies. Since sipuleucel-T became the first cancer vaccine licensed in the United States [1], [2], there has been greatly renewed interest in cancer vaccines [3]–[5]. Thus, novel tumor antigens specific to particular cancer types are of great interest.

CD8^+^ CTLs can recognize and specifically kill tumor cells expressing peptides from tumor-associated antigens presented by MHC class I molecules. Therefore, most current cancer immunotherapy strategies focus on induction of CTLs that lyse tumor cells [6]. Antigen-specific cancer immunotherapy often relies on identification of epitopes expressed by cancer cells that can be used as targets for CD8^+^ T cells. However, the natural CTL epitopes of cancers are not always optimal because the CTL repertoire against high-affinity epitopes is often tolerized [7]. Epitope enhancement, by means of modification of the AA sequence of epitopes, was developed to improve the efficacy of vaccines primarily through increasing affinity of peptides for MHC molecules [3], [8]. Peptide vaccines have recently shown considerable promise in clinical trials [9].

Discovering tumor-specific antigens is critical to the development of effective cancer immunotherapy [5]. Recently, a novel tumor-associated antigen, POTE, was identified from a variety of human cancers [10], [11]. This tumor antigen is called POTE because it was at first found to be expressed in normal prostate, ovary, testis, and placenta tissues, as well as in prostate cancer [10]. The POTE gene family was found dispersed among eight different chromosomes (2, 8, 13, 14, 15, 18, 21, and 22) with different length of mRNAs [12]. Nevertheless, the POTE cDNA sequence among various chromosomes is highly homogeneous with the divergence less than 10% [11]. Subsequent studies revealed that POTE genes were expressed not only in prostate cancer but also in a wide variety of human malignancies, including breast, colon, lung, ovary and pancreas [11]. There are distinct patterns of expression of POTE in normal tissues and cancers [11]. Among the various cancers, the POTE paralogs on chromosome 2 (POTE-2γ) are the most frequently expressed.

Because POTE mRNA is detectable only in a limited number of normal human tissues (prostate and testis in the male, and ovary and placenta in the female), the POTE protein is considered as a member of the cancer-testis antigen family [11]. Expression of cancer-testis antigens in the placenta or testis should not lead to T-cell activation because of the very low expression of MHC class I molecules in these tissues [13], [14]. For cancer patients, some reactivity against prostate or breast tissue is acceptable, as neither are vital tissues. Moreover, for breast cancer or prostate cancer patients, the prostate in the male and breast in the female should have been surgical resected or irradiated. The concerns of autoimmune reaction in both tissues would not be an impediment in the treatment of life-threatening cancer. Therefore, the POTE antigen should be an attractive target for the immunotherapy of these cancers.

In this study, we first determined HLA-A2-restricted epitopes from POTE (2γ), and their immunogenicities were tested in AAD transgenic mice that have a chimeric MHC class I molecule composed of α1 and α2 domains from HLA-A2 and α3 domain from D^d^ [15]. Next, we enhanced their binding to HLA-A2.1 molecules by epitope enhancement to make the epitopes more immunogenic, without losing the ability of specific CD8^+^ T cells to recognize the wild type epitope to a significant degree. Finally, we confirmed the modified epitopes induced CTLs that killed human cancer cells.

## Materials and Methods

### Animals

AAD mice [15] expressing a chimeric HLA-A2.1 transgene with the α1 and α2 domains from HLA-A2.1 and the α3 domain from H-2D^d^, to allow binding to mouse CD8, on a C57BL/6 background, were used in these experiments. Parts of the experiments were repeated in HHD-2 mice (a gift from Dr. François Lemmonier, Institut Pasteur), which express chimeric human HLA-A2.1 with the covalent human β2m, without any murine class I molecule [16]–[18]. Mice were bred and housed in appropriate animal care facilities. All procedures with animals were conducted in accordance with the institution’s approved protocols.

### Peptides

Peptides in this study were synthesized on a Model Symphony Peptide Synthesizer (Perkin-Elmer, Boston, MA) using conventional fluorenylmethoxycarbonyl (f-MOC) chemistry and cleaved from the resin by trifluoroacetic acid. The purity and molar concentration were analyzed by reverse phase high-performance liquid chromatography on a C18 column using a gradient of 0.1% trifluoroacetic acid in water and 0.1% trifluoroacetic acid in acetonitrile and further purified by preparative reverse-phase high-performance liquid chromatography using a similar gradient.

### Cell Lines

The T2 cell line is deficient for TAP1 and TAP2 transporter proteins and expresses low levels of HLA-A2 [19]. The C1R.AAD cell line is derived from the human B lymphoblastoid cell line HMYC1R transfected with the HLA chimeric molecule containing α1 and α2 domains from human HLA-A2.1 and α3 from mouse H-2D^d^, a gift from Dr. V. Engelhard (University of Virginia, Charlottesville, VA) as previously described [15]. The C1R.A2.1 cell line (a gift of Dr. William Biddison, NINDS, NIH) is also derived from HMYC1R transfected with HLA-A2.1 [20], [21]. C1R.AAD and C1R.A2.1 cells were maintained in complete medium consisting of 10% FCS-RPMI 1640, 1 mM sodium pyruvate, nonessential amino acids (Biofluid, Rockville, MD), 4 mM glutamine, 100 U/ml penicillin, 100 µg/ml streptomycin, and 50 µM 2-ME. NCI-H522 (POTE^+^/HLA-A2^+^) was maintained in complete medium. HTB-19 (POTE^+^/HLA-A1^+^) was maintained in Eagle’s minimum essential medium (EMEM) with 5% FCS. MDA-MB-231 (POTE^–/^HLA-A2^+^) was maintained in 10% FCS-DMEM.

#### T2 binding assay

Peptide binding capacity to the HLA-A2 molecule was measured by using the T2 cell line according to a protocol described previously [19], [22]. T2 cells (3 × 10^5^ cells/well) were incubated overnight in 96-well plates with culture medium (1∶1 mixture of RPMI 1640/Eagle-Hank’s amino acid containing 2.5% fetal bovine serum, 100 U/ml penicillin, and 100 µg/ml streptomycin) with 10 µg/ml β2-microglobulin (Sigma) and different titrated concentrations of peptides (starting from 100 µM with 2-fold serial dilution) as shown in the figures to determine the concentration giving a 50% increase in binding (see below). Cells were washed once with cold HBSS containing 0.1% BSA and incubated for 30 min at 4°C with anti-HLA-A2.1 monoclonal antibody (clone BB7.2) (1∶40 dilution from hybridoma supernatant). After washing, cells were stained with FITC-labeled goat anti-mouse immunoglobulin (BD, San Jose, CA), and the level of HLA expression was measured by flow cytometry. HLA-A2 expression was quantified as fluorescence index (FI) according to the following formula: FI = (geometric mean fluorescence intensity with peptide - geometric mean fluorescence intensity without peptide)/geometric mean fluorescence intensity without peptide. Background fluorescence without BB7.2 was subtracted for each individual value. To compare the different peptides, FI_0.5_, the peptide concentration that increases HLA-A2.1 expression by 50% over no peptide control background, was calculated from the titration curve for each peptide. It should be noted that the FI_0.5_ provides a relative measure of peptide affinity or potency among peptides compared side by side, but cannot be taken as a thermodynamic affinity.

### Immunizations

AAD or HHD-2 mice were immunized s.c. in the base of tail with 100 µl of an emulsion containing 1∶1 incomplete Freund’s adjuvant (Sigma) and PBS with peptides and cytokines (50 nmol of CTL epitope, 50 nmol of hepatitis B virus core 128–140 helper epitope, 5 µg of mouse interleukin 12, and 5 µg of mouse granulocyte macrophage colony-stimulating factor). Cytokines were purchased from Peprotech (Rocky Hill, NJ). Mice were boosted 2 weeks later, and the spleens were removed 2 weeks after the boost. For all experiments, groups of three mice were used.

### Interferon-γ ELISPOT Assay

The numbers of IFN-γ secreting cells were determined by an ELISPOT kit (Mouse IFN-gamma ELISpot SixPak; R&D, Minneapolis, MA) or by a set of anti-mouse IFN-γ coating (AN18) and detecting (R4–6A2) monoclonal antibodies (Mabtech) according to the manufacturer’s instructions. Two weeks after the last immunization, spleen cells were plated on antibody-coated plates at 200,000, 100,000 and 50.000 cells/well and stimulated with 200,000 peptide-pulsed naïve spleen cells/well with indicated concentration of peptides for 2 hours. After incubation overnight at 37°C, the plates were washed and detecting antibody was added. An ELISPOT blue color module (R&D) was used for color development. The spots were read by an ELISPOT reader (AID). All samples were analyzed in triplicate.

### *In vitro* Stimulation and Cytotoxicity Assay

Splenocytes from the immunized mice were restimulated with peptide-loaded splenocytes (1 µM). On day 1 and day 5, Rat T Stim (BD Bioscience) was added as a source of IL-2. One week later, CTL activity was measured by using a 4-h ^51^Cr releasing assay. Target cells (10^6^) were pulsed with peptides in 200 µl of complete T cell medium and 150 µCi of ^51^Cr for 2 h, washed three times, and added at 3000 cells/well to the 96-well round-bottom plates with different E/T ratios. The percentage of specific ^51^Cr release was calculated as 100 X [(experimental release - spontaneous release)/(maximum release - spontaneous release)]. Spontaneous release was determined from target cells incubated without effector cells, and maximum release was determined in the presence of 5% triton-X. C1R.AAD or C1R.A2.1 cell lines with or without peptides or human cancer cells without peptides were used as targets. C1R.AAD or C1R.A2.1 cell lines do not express any other MHC molecule, either class I or class II, besides HLA-A2, so they are not subject to concerns about allo- or xenoreactivity. Human cancer cells were preincubated with 1000 ng/ml human IFN-γ for 72 h prior to the assay [23].

### HLA-A2/peptide Tetramer Complexes

Tetrameric MHC class I/peptide complexes obtained from the NIH Tetramer Facility were synthesized as described previously [24], [25]. Briefly, purified single-chain HLA-A2 molecules were synthesized by a prokaryotic expression system (pET; R&D Systems, Minneapolis, MN). The heavy chain was modified containing the Bir-A enzymatic biotinylation site. Single-chain heavy chain-β2-microglobulin and peptide were refolded by dilution into a redoxshuffling buffer system. The refolded product was biotinylated by Bir-A in the presence of biotin ligase (Avidity, Denver, CO). Streptavidin-Phycoerythrin conjugate (Jackson ImmunoResearch, West Grove, PA) was added in a 1∶4 molar ratio. Per-CP labeled anti-mouse CD8 antibody (BD Pharmingen) and TCR Vβ screening panel (BD Pharmingen) were used to detect the TCR repertoire of the CTLs.

### Epitope Enhancement

To improve binding of the POTE epitopes to HLA-A2, we examined the sequences for primary and secondary anchor residues that might be suboptimal, based on defined primary and secondary anchors [26], [27], and synthesized peptides with single AA substitutions to remedy these deficiencies. These were then tested empirically as described in Results for binding and immunogenicity and for the activity of the CTL induced.

### Ethics Statement

All experiments with mice were approved by the Animal Care and Use Committee of National Cancer Institute, NIH and of Taipei Veterans General Hospital.

## Results

### Prediction of HLA-A2.1-restricted Epitopes

The prototype sequence of POTE has 584 AA residues (Accession number AY172978, ref [12]). Five 9-mer peptides were first selected based on AA anchor residues that determine binding to HLA-A2 molecules and three predictive algorithms [22], [26], [28]–[32]. The Parker score is based on a predicted half-time of dissociation to HLA class I molecules using a matrix of AAs at each position in the sequence based on known binding peptides, and a score of higher than 100 is suggested as a potential binder. The second algorithm was developed by Dr. Zhiping Weng, which is based on linear programming for predicting HLA-A2 binding peptides (http://zlab.bu.edu/SMM/). A In(IC50) lower than 8 is predicted to be an HLA-A2 binder. The SYFPEITHI score was developed by Hans-Georg Rammensee's lab [29] (http://www.syfpeithi.de/). A SYFPEITHI score higher than 21 predicts a potential HLA-A2 epitope. If the sequence was suggested as a binder by any one of the predictive algorithms, the peptide was used for following study. As shown in Table 1, predictions were not completely consistent among the three algorithms.

**Table 1:** List of predicted HLA-A2 binders of POTE protein.

Position	Sequence	K Parker score	Z Weng ln(IC50)	SYFPEITHI score	FI_0.5_
POTE 222–230	VLMLLEHGA	31.249	3.10	19	42.4 µM
POTE 252–260	KLMAKALLL	276.643	6.10	24	1.6 µM
POTE 272–280	GLTPLLLGV	159.970	5.30	29	0.9 µM
POTE 323–331	LLLEQNVDV	1793.677	4.91	28	0.8 µM
POTE 553–561	KILEEIESV	572.255	5.91	29	5.6 µM

### Determine the Binding Affinity of POTE Peptides to HLA-A2 Molecules

**Figure 1:**
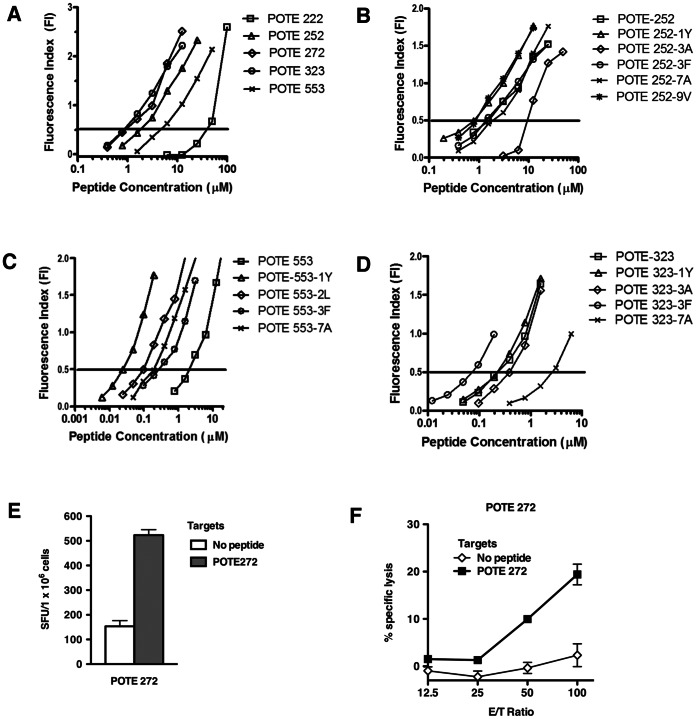
T2 binding assays and immunogenicity study. (A). The HLA-A2 binding affinity of the 5 predicted POTE epitopes was confirmed by T2 binding assay. The calculated FI_0.5_ for POTE 222, 252, 272, 323 and 553 were approximately 42.4 µM, 1.6 µM, 0.9 µM, 0.8 µM and 5.6 µM, respectively. Binding affinity of the substituted POTE 252 peptides (B), the substituted POTE 553 peptides (C) or the substituted POTE 323 peptides (D) to HLA-A2 molecules. Each assay was performed in triplicate, and data in this figure are representative of two experiments with similar results. Immunogenicity of the POTE 272 epitope in AAD mice (E). Mice were immunized with 50 nmol of POTE 272 in 100 µl of emulsion, and targets were pulsed with 1.0 µM POTE272. Spots were counted by an ELISPOT reader (AID ELISPOT reader system). Figures show numbers of spots per million cells. (F) CTL reactivity on POTE 272 peptide. In a 4 hour ^51^Cr release assay, CIR.AAD cells were pulsed with 1.0 µM POTE 272 peptide and labeled with ^51^Cr. After washing three times, target cells were mixed with different numbers of effector cells and then cultured for 4 hours before harvesting.

The binding affinity of the predicted peptides with HLA-A2 molecule was measured by the T2 binding assay. As shown in Figure 1A, POTE 323-331 and POTE 272–280 showed the strongest binding affinity for the HLA-A2 molecule with an FI_0.5_ of 0.8 µM and 0.9 µM, respectively. POTE 252–260 and POTE 553–561 were intermediate binders with FI_0.5_ of 1.6 µM and 5.6 µM, respectively. Because POTE 222–230 is a weak binder with an FI_0.5_ around 42.4 µM, this sequence has little chance to bind and be presented by HLA-A2 molecules to CD8^+^ T cells. Good and intermediate binders were chosen for immunogenicity studies in HLA-A2 transgenic mice.

### Epitope Enhancement to Increase the Binding Affinity of POTE Peptides for HLA-A2 Molecules

T cells specific for self-peptides with good capacity to bind with MHC class I molecule are frequently negatively selected [7]. Tumor-associated peptides displaying intermediate MHC class I binding affinities might be preferable as vaccine candidates. In addition, the MHC class I binding and immunogenicity of such intermediate affinity binders can be improved by substitution of primary or secondary anchor AA with more optimal binding ones, a procedure named epitope enhancement. We made peptides with AA substitutions to insert known good primary or secondary anchor residues when these were not already present, based on previous reports [26], [27]. Therefore, we made such modifications of intermediate binders, POTE 252 and POTE 553, and again determined the binding affinity with HLA-A2 molecules by the T2 binding assay. Besides the intermediate binders, we also made AA substitutions in one of the best binders, POTE 323, to examine whether modification could further improve its binding affinity to HLA-A2 molecules. As shown in Table 2, AA substitutions in primary (position 2 and the C terminus) or secondary (position 1, 3 and 7) anchor residues for HLA-A2 molecules were made if the native residues were not already optimal [22], [26], [28], [30]. We did not try to enhance POTE 272 as we judged that it was unlikely to be substantially improved.

**Table 2:** List of modified peptides from POTE protein.

Peptide	Sequence	FI_0.5_
POTE 252	KLMAKALLL	1.5 µM
POTE 252-1Y	YLMAKALLL	0.9 µM
POTE 252-3A	KLAAKALLL	16.9 µM
POTE 252-3F	KLFAKALLL	1.5 µM
POTE 252-7A	KLMAKAALL	4 µM
POTE 252-9V	KLMAKALLV	0.8 µM
POTE 553	KILEEIESV	2.6 µM
POTE 553-1Y	YILEEIESV	0.024 µM
POTE 553-2L	KLLEEIESV	0.1 µM
POTE 553-3F	KIFEEIESV	0.3 µM
POTE-553-7A	KILEEIASV	0.2 µM
POTE 323	LLLEQNVDV	0.3 µM
POTE 323-1Y	YLLEQNVDV	0.25 µM
POTE 323-3A	LLAEQNVDV	0.4 µM
POTE 323-3F	LLFEQNVDV	0.09 µM
POTE 323-7A	LLLEQNADV	2.78 µM

Five modified peptides were generated based on the intermediate binder, POTE 252. As showed in Figure 1B, both POTE 252-9V and POTE 252-1Y had better binding affinity to HLA-A2 molecules than the wild type, POTE 252. The FI_0.5_ of POTE 252-9V and POTE 252-1Y was 0.8 µM and 0.9 µM, respectively. Both were nearly 2-fold lower (higher affinity) than that of the POTE 252 peptide.

Four substituted peptides were made for another intermediate binder, POTE 553. As shown in Figure 1C, all of the modified peptides, POTE 553-1Y, POTE 553-2L, POTE 553-7A and POTE 553-3F had better binding affinity to HLA-A2 molecules than the wild type POTE 553 peptide. The FI_0.5_ values of POTE 553-1Ywas 0.024 µM.

Four peptides were also modified from the best binder, POTE 323. As shown in Figure 1D, only POTE 323-3F could enhance the binding affinity to HLA-A2 molecules, with the FI_0.5_ of 0.09 µM, approximately 3-fold better than the wild type peptide.

### Immunogenicity of a Good HLA-A2.1 Binder, POTE 272

To test the immunogenicity of the POTE 272 peptide, groups of 3 AAD transgenic mice were immunized with POTE 272 peptide subcutaneously. Two weeks after the booster, the splenocytes from 3 mice were pooled to measure their immunogenicity by *ex vivo* IFN-γ ELISPOT assay and by CTL assay after one week *in vitro* stimulation. As shown in Figure 1E, POTE 272 induced significant IFN-γ responses following POTE 272 stimulation. After one week *in vitro* stimulation, CTLs induced with POTE 272 lysed target cells pulsed with POTE 272 (Figure 1F). Therefore, POTE 272 is an immunogenic epitope in the POTE protein. However, CD8^+^ T cells specific to self-antigen with good binding affinity may be negatively selected or self-tolerant, so POTE 272 may not be a good candidate as a cancer vaccine for the POTE protein, although this remains to be tested.

### Immunogenicity of the Wild Type and Enhanced POTE 252 Epitopes and CD8^+^ T-Cell Cross-reactive Responses

**Figure 2:**
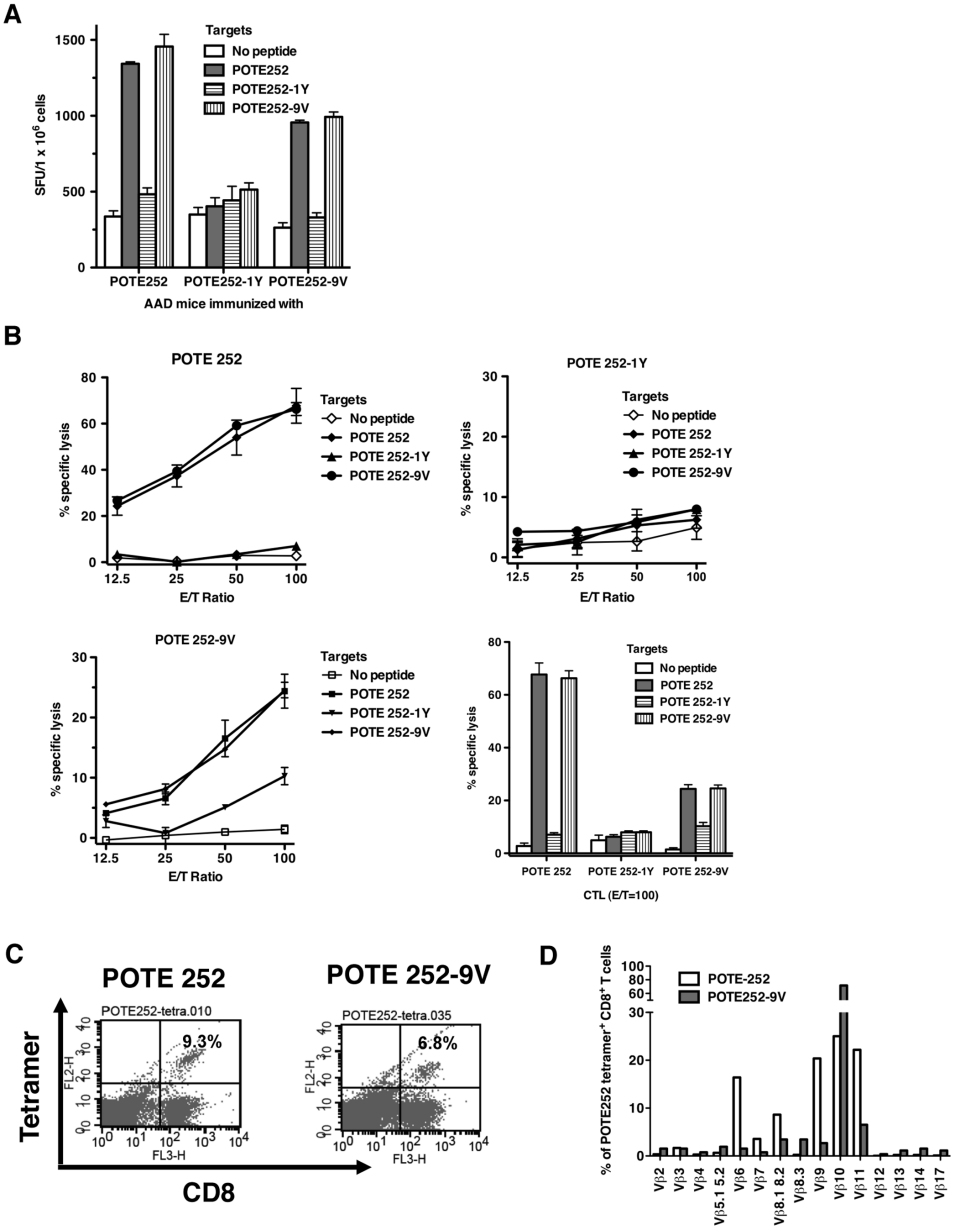
Immunogenicity of the wild type and enhanced POTE 252 epitopes in AAD mice. (A) Mice were immunized with 50 nmol of POTE 252 in 100 µl of emulsion, and targets were pulsed with 1.0 µM POTE252. Spots were counted by an ELISPOT reader (AID ELISPOT reader system). Figures show numbers of spots per million cells. (B) CTL cross-reactivity on each peptide. Two weeks after the second boost, pooled spleen cells from three mice were restimulated with irradiated splenocytes pulsed with 1.0 µM each peptide for 7 days. Then a 4 hour ^51^Cr release assay was performed. (C) HLA-A2/peptide tetramer staining and (D) TCR repertoire determination for POTE 252- and POTE 252-9V-induced CTLs in HHD-2 mice.

Both POTE 252-9V and POTE 252-1Y had better binding affinity to HLA-A2 molecules than the POTE 252 (Figure 1B). As shown in Figure 2A, both POTE 252 and POTE 252-9V induced a significant number of IFN-γ spots with cross-reactivity to both POTE 252 and POTE 252-9V. Although POTE 252-1Y had a better binding affinity to HLA-A2 molecules than POTE 252, no significant IFN-γ responses were detected in the assay even in the presence of POTE 252-1Y peptide. After 1-week of in vitro stimulation with cognate peptide, CD8^+^ CTLs induced with both wild type POTE 252 and enhanced epitope POTE 252-9V lysed target cells pulsed with the wild type POTE 252 as well as POTE 252-9V. This result suggested that the TCRs of CTLs generated in mice immunized with either wild type or enhanced peptide could recognize the wild type peptide (POTE 252)/MHC class I complex (Figure 2B).

### HLA-A2 Tetramer Stain and Analysis of TCR Vβ Repertoire

The CTLs induced by POTE 252-9V were the most strongly cross-reactive with wild type POTE antigen. We further compared the response and the TCR repertoire usage of the POTE-specific CD8^+^ T cells from HHD-2 mice immunized with POTE 252 and 252-9V. As shown in Figure 2C, 9.3% versus 6.8% of the CD8^+^ T cells were tetramer-positive in POTE252 and POTE 252-9V immunization groups, respectively. By comparing the TCR repertoire of the CD8^+^tetramer^+^ T cells between the two groups, the data (Figure 2D) showed that the CTLs induced by POTE 252 were positive for Vβ 6, 7, 8.1, 8.2, 9, 10, 11, with most being either Vβ 6, 9, 10 or 11, whereas the CTLs induced by POTE 252-9V were positive for Vβ 8, 9, 10, and 11, but were overwhelmingly dominated by about 80% positive for Vβ10 and only a small fraction expressing the others. Thus, the TCR repertoires of the CD8^+^tetramer^+^ T cells were distinct between the wild type and enhanced POTE peptide immunized mice.

### Immunogenicity of the Wild Type and Enhanced POTE 553 Epitopes and CD8^+^ T-Cell Cross Responses

**Figure 3:**
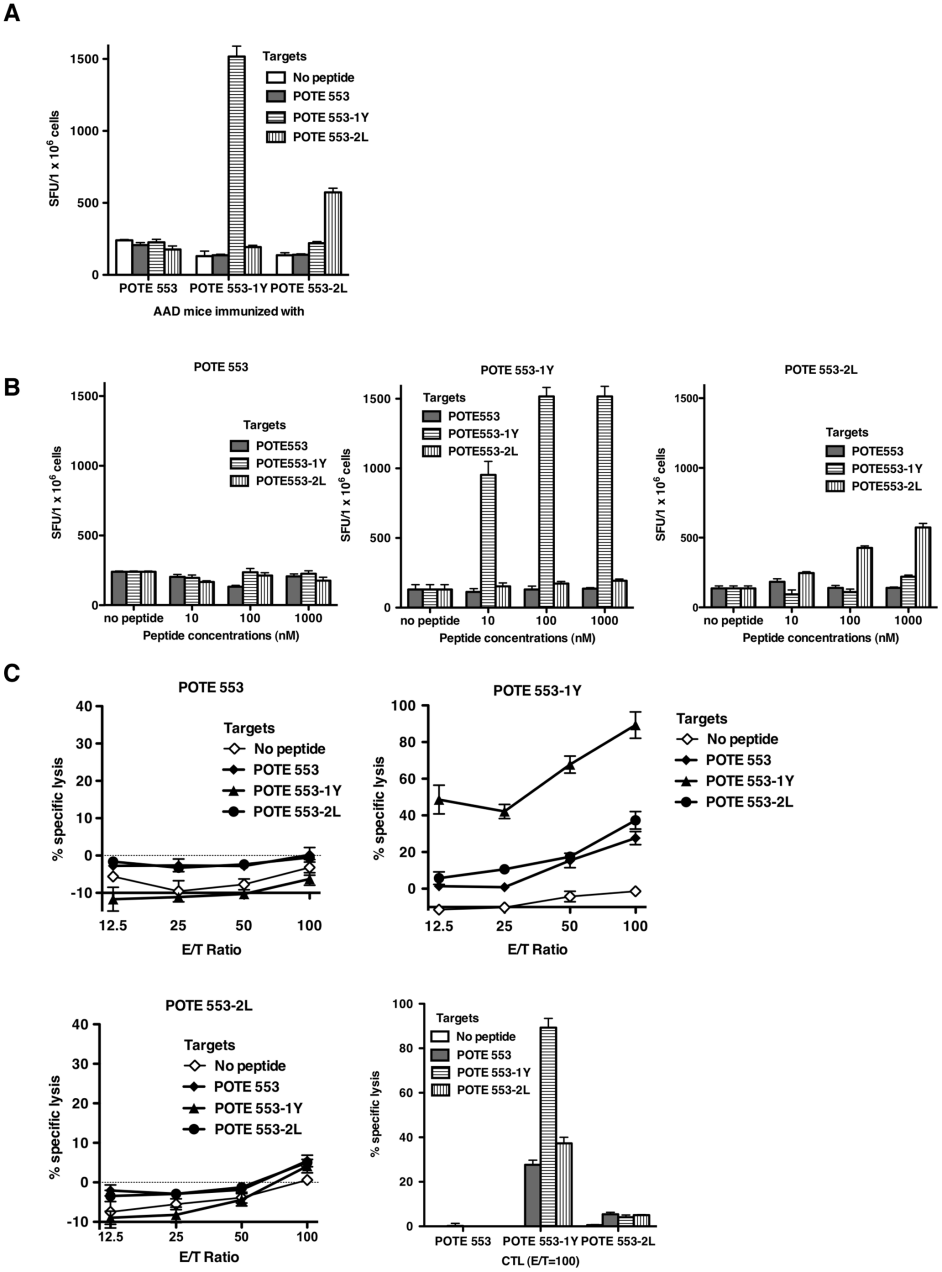
Immunogenicity of the wild type and enhanced POTE 553 epitopes in AAD mice. AAD mice were immunized s.c. with a mixture of peptide and cytokines in adjuvant described in the Methods section. (A,B) Two weeks after the second boost, splenocytes pooled from three mice were restimulated with splenocytes of naïve AAD mice pulsed with 10 nM to 1000 nM of each peptide at different E/T ratios (1000 nM was used in panel A). Spots were counted by an ELISPOT reader (AID ELISPOT reader system). Figures show numbers of spots per million cells. (C) CTL cross-reactivity on each peptide. In a 4 hour ^51^Cr release assay, CIR.AAD cells were pulsed with 1.0 µM each peptide and labeled with ^51^Cr. After washing three times, target cells were mixed with different numbers of effector cells and then cultured for 4 hours before harvesting.

All the substituted POTE 553 peptides showed better binding affinity to HLA-A2 molecules than the POTE 553 (Figure 1C). We chose the two best binders among these enhanced peptides, along with the wild type peptide, to test the immunogenicities and cross-reactive CTL responses. As shown in Figure 3A and B, POTE 553 did not induce any significant IFN-γ responses at any peptide concentration used for stimulation. After one week *in vitro* stimulation, there was likewise not any significant detectable target cell lysis (Figure 3C). Therefore POTE 553, as an intermediate HLA-A2 binder, is not immunogenic. Both POTE 553-1Y and POTE 553-2L induced some level of IFN-γ responses only after stimulation with the same peptide (Figure 3A, B). In Figure 3B, the target cells were pulsed with different concentrations of peptide to demonstrate that the enhanced peptide could induce high avidity T cells to kill target cells in the presence of low antigen concentrations. According to the *ex vivo* IFN-γ ELISPOT assay, no cross-reactive response to wild type peptide was detected in the CTLs induced by POTE 553-1Y or POTE 553-2L peptides. However, after one week *in vitro* stimulation, CD8^+^ CTLs induced with the enhanced epitope POTE 553-1Y lysed target cells pulsed with not only POTE 553-1Y, but also POTE 553 and POTE 553-2L peptides, suggesting that the TCRs of the CTLs recognized the POTE 553/MHC class I complex to some extent (Figure 3C). From this point of view, POTE-553-1Y might be a possible alternative cancer vaccine against the POTE antigen. Although POTE 553-2L can induce certain IFN-γ responses after stimulation with the same peptide (Figure 3A, B), CD8^+^ CTLs induced with POTE 553-2L did not lyse target cells pulsed with POTE 553-2L (Figure 3C).

### Immunogenicity of the Wild Type and Enhanced POTE 323 Epitopes and CD8^+^ T-Cell Cross-reactive Responses

**Figure 4:**
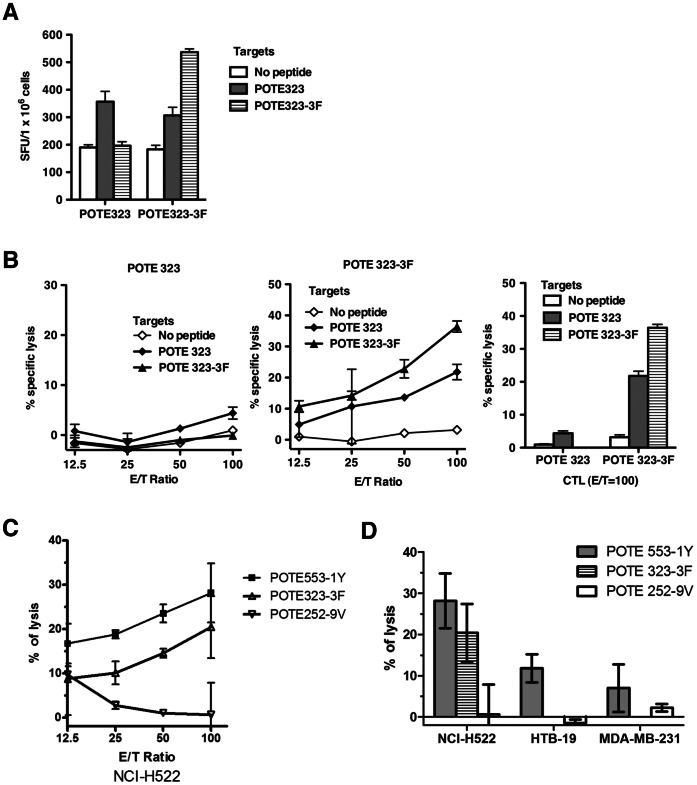
Immunogenicity of the wild-type and enhanced POTE323 epitopes and anti-tumor cytotoxicity induced by enhanced epitopes. AAD mice were immunized s.c. with 50 nmol of peptide in 100 µl of emulsion as described in the Methods section. (A) Direct ex vivo IFN-γ ELISPOT assay using target cells pulsed with 1000 nM peptide. Figures show numbers of spots per million cells. (B) CTL cross-reactivity on POTE 323 and POTE 323-3F peptides. (C) Anti-tumor cytotoxicity against a POTE-expressing human lung cancer cell line NCI-H522 induced by POTE 553-1Y, 323-3F and 252-9V. HHD-2 mice were immunized with 50 nmol of the peptide indicated in 100 µl of emulsion and restimulated for 7 days with 1000 nM peptide before being tested for ability to lyse the tumor cells. (D) Summary of the anti-tumor cytotoxicity in POTE-expressing and non-POTE-expressing human tumor cells. NCI-H522 is a human lung cancer line expressing both POTE and HLA-A2, whereas HTB-19 is a human mammary carcinoma that expresses POTE but not HLA-A2, and MDA-MB-231 is a human mammary carcinoma cell line expressing HLA-A2 but not POTE. Killing of only the first of these shows the specificity for POTE in combination with HLA-A2. (Negative values are due to experimental ^51^Cr release slightly below spontaneous release with no effector cells, within experimental error, and so should be viewed as equivalent to zero).

POTE 323-3F was the only modified peptide with better HLA-A2 binding affinity than wild type POTE 323 (Figure 1D). As shown in Figure 4A, both POTE 323 and POTE 323-3F induced some IFN-γ responses after stimulation with the identical peptide. Even though the POTE 323 peptide was the best HLA-A2 binder within the POTE sequence, only marginal IFN-γ responses (less than 2-fold over background level) were detected by the ELISPOT assay. After one week *in vitro* stimulation, CTLs induced by POTE 323 failed to lyse target cells pulsed with POTE 323. This negative CTL response might be due to the loss of a substantial portion of responding cells with high avidity that could have been killed during the incubation with a relative high concentration of peptide (1.0 µM). POTE 323-3F induced significant IFN-γ spots under POTE 323-3F stimulation, and some degree of IFN-γ response was also detected after stimulation with wild type POTE 323 epitope. After *in vitro* stimulation with the cognate peptide, CD8^+^ CTLs induced with the enhanced epitope POTE 323-3F lysed target cells pulsed with the POTE 323-3F, as well as wild type POTE 323, suggesting that the TCRs of the CTLs could recognize the wild type peptide (POTE 323)/MHC class I complex (Figure 4B). POTE 323-3F was more immunogenic in inducing CTLs specific for the wild type epitope than was the wild type epitope itself. Therefore, POTE 323-3F might be an excellent vaccine candidate to target tumor cells expressing POTE.

### CTLs Induced by Modified POTE Peptides Against Human Cancer Cells

To serve as candidate tumor vaccines, induction of cytotoxicity to kill human cancer cells is essential. Three human cancer cell lines, NCI-H522 (human non-small cell lung cancer cell, POTE^+^/HLA-A2^+^), HTB-19 (human mammary carcinoma, POTE^+^/HLA-A1^+^) and MDA-MB-231 (human breast cancer cell, POTE^–/^HLA-A2^+^) were chosen as target cells in this study. As shown in Figure 4C & 4D, although POTE 252-9V is the most immunogenic, the CTLs induced by the modified peptide did not kill POTE-expressing cancer cells, even at high E/T ratios. This suggests that POTE 252 may not be correctly processed and presented on HLA-A2 in these tumor cells. In contrast, the CD8^+^ T cells induced by both POTE 553-1Y and POTE 323-3F immunization and restimulation could produce significant cytotoxicity to lyse NCI-H522 cells. Killing of only the tumors that express both POTE and HLA-A2 and no significant killing above background of tumors expressing either one alone confirms the specificity of the killing by these CTL for POTE and HLA-A2. These results prove that at least the POTE 553 and 323 epitopes are naturally endogenously processed in human tumor cells and presented on human HLA-A2, and also that the epitope-enhanced peptides POTE 553-1Y and 323-3F can induce CTL that recognize the corresponding naturally processed wild type POTE epitopes on human cancer cells.

## Discussion

Since a cancer vaccine was licensed by the FDA for the first time in the United States [1], [2], [33], there has been greatly renewed interest in cancer vaccines and identifying useful tumor antigens [3]–[5]. Epitope enhancement by sequence modification was designed to improve such vaccines by increasing the immunogenicity of cancer epitopes that are often derived from self antigens [3], [6]–[8], [34], [35]. Despite occasional findings that some modified peptides may not always be recognized by tumor-reactive T cells [36], sequence modifications both within the epitope and in flanking residues have been useful in increasing immunogenicity in many cases, by affecting MHC binding or antigen processing [3], [7], [23], [37], [38]. For these reasons, we have undertaken identification and epitope enhancement of epitopes from the novel tumor antigen POTE presented by the most common human HLA class I molecule, HLA-A2 (HLA-A*0201).

POTE is a newly found tumor antigen expressed in breast, prostate, colon, lung, ovarian and pancreatic cancers [10], [11]. Because only a limited number of normal tissues can express POTE antigen, POTE could be used as a unique target protein for cancer immunotherapy for a variety of cancers. In particular, if autoimmunity were induced, the normal tissues against which any autoimmune response to POTE might be generated, such as prostate in prostate cancer or breast in breast cancer, would be ones that would be ablated by surgery or radiation in those diseases and are not vital tissues. In addition, as nearly half of the American population is HLA-A2^+^, POTE peptides described here may serve as an effective cancer vaccine for those patients.

In this study, we defined peptide immunogens from the POTE protein and examined the potential of substituted peptides as candidates for the immunotherapy of lung, breast, or other cancers. To define HLA-A2 epitopes from the POTE protein, 9-mer peptides were predicted based on AA anchor residues that determine binding to HLA-A2 molecules using three predictive algorithms [22], [28], [29]. Based on a T2 binding assay [19], it appears that none of the predictive algorithms can predict perfectly the rank order of binding affinity to HLA-A2 molecules. Therefore, it appears that these algorithms can be used only for preliminary mass screening. For the POTE protein, the SYFPEITHI score best correlated with the experimental binding data.

POTE is composed of over 500 AA residues, among which we defined four 9-mer peptides that had a significant binding affinity to HLA-A2 molecules. Most of the tumor antigens are self-antigens, including the POTE protein. CD8^+^ T cells specific to self-antigens are usually negatively selected in the thymus during T cell development, especially those with good MHC class I binding affinity (self tolerance). To use a self-antigen as a cancer vaccine target, one possibility is to develop enhanced epitopes that are potentially more immunogenic and against which CD8^+^ T cells specific to the enhanced epitopes may not be deleted or negatively selected [7], [39]. More importantly, the TCR specific to enhanced peptide/MHC class I complex will be useful only if it is able to recognize the wild type peptide/MHC class I complex expressed by the tumor cells. Although there are cases in which wild type vaccine peptides are more effective than modified peptides [40], probably because the altered peptide induces a repertoire that only partially cross-reacts with the wild type epitopes in some cases, here we have verified that the T cells specific for the epitope-enhanced peptide kill human cells expressing wild type POTE.

We first made AA substitutions for intermediate HLA-A2 binders, POTE 252 and POTE 553. Substitution of Leu at position 9 with Val, and Lys at position 1 with Tyr in POTE 252 moderately improved the peptide binding affinity to HLA-A2 molecules. This could be explained by the fact that Val is the optimal AA for the C-terminal anchor residue for HLA-A2-binding peptides, and Tyr in position 1 was reported to stabilize the binding of peptide/MHC complex [7], [32], [41], [42]. The immunogenicity of the wild type and enhanced peptides was compared by using AAD and HHD-2 transgenic mice. These mice have been shown to be good predictors of human T cell epitopes [15], [17], [39], [43]. CD8^+^ CTLs induced with wild type POTE 252 or enhanced POTE 252-9V peptides could recognize cross-reactive POTE252 or POTE252-9V/MHC complexes. The range of TCR recognition induced by both the peptides appeared to overlap substantially in ELISPOT and CTL assays. However, the TCR Vβ repertoires of the tetramer^+^CD8^+^ T cells were distinct between the POTE252- and POTE 252-9V-induced CTLs. However, the failure of CTL specific for this epitope to kill human tumor cells expressing POTE and HLA-A2 suggests that it may not be processed well endogenously and therefore may not be a good target antigen.

For another intermediate HLA-A2 binder, POTE 553, substitution of Lys at position 1 with Tyr, Ile at position 2 with Leu, Leu at position 3 with Phe, and Glu at position 7 with Ala greatly improved the peptide binding affinity to HLA-A2 molecules. Improvements in HLA-A2 binding may be explained by (i) the fact that Leu at position 2 is an optimal HLA-A2 anchor residue [30]: (ii) Asp, Glu, Arg, Lys and His at position 7 are associated with poor binding to HLA-A2 [26]. Leu at position 3 did not have an adverse effect on HLA-A2 binding affinity, but Phe at position 3 can improve its binding affinity [39]. In the immunogenicity studies, although POTE 553 is not immunogenic, the enhanced epitope POTE 553-1Y showed greatly improved immunogenicity. CD8^+^ T cells raised with this epitope can recognize a range of cross-reactive peptide/MHC complexes. Several studies reported that tyrosine substitution at position 1 of an HLA-A2-restricted CTL epitope could increase its binding affinity and did not interfere with TCR interaction [32], [41], [42]. We also found that CD8^+^ T cells induced by POTE-553-1Y cross-reactively recognized wild type POTE 553 peptide/MHC class I complex as well as POTE-expressing human cancer cells, NCI-H522. Thus, this epitope is endogenously processed for class I HLA presentation in human tumor cells, and CTL to the enhanced peptide recognize the wild type naturally processed peptide.

POTE 323 is the best HLA-A2 binder among the predicted epitopes. No AA residues with adverse effects on HLA-A2 binding affinity existed in its sequence. Nevertheless, substitution of Leu at position 3 with Phe further improved the binding affinity to HLA-A2 molecules. Although POTE 323 was the best HLA-A2 binder among the wild type peptide tested, its immunogenicity was not consistent with its binding affinity in the mouse study. One potential explanation was the possibility that mouse POTE and human POTE share a common sequence in this region, so that mouse CD8^+^ T cells specific to POTE 323 might be negatively selected. However, we can exclude this possibility because the POTE protein is expressed only in primates. The enhanced epitope POTE 323-3F has not only higher binding affinity but also improved immunogenicity. More importantly, CD8^+^ T cells induced by POTE 323-3F cross-reactively recognized POTE 323/MHC class I complexes on both peptide-pulsed targets and human tumor cells naturally expressing POTE. Therefore POTE 323-3F could be a very promising vaccine target for cancers expressing POTE, because the low immunogenicity of the wild type peptide suggests that it may not be tolerogenic either, so an enhanced peptide that elicits T cells cross-reactive with wild type POTE 323 is also effective against NCI-H522, the POTE-expressing tumor cells. This finding confirms that POTE323 is naturally processed and presented on human tumor cells in association with HLA-A2. Furthermore, the fact that the 323 peptide induced T cells that make IFN-γ but showed poor lytic activity, whereas the 323-3F peptide induced T cells with slightly improved IFN-γ production but much greater lytic activity suggests that the POTE 323-3F peptide induces a response that is improved in quality as well as quantity and can kill human tumor cells expressing endogenously processed wild type POTE.

Both POTE 553-1Y and POTE 323-3F peptides are promising candidates for cancer vaccines that can induce CD8^+^ T cells to kill POTE-expressing human cancer cells. Since we could not immunize humans in this study, it is well established that the TCR repertoires in both mouse and humans are so broad that they are not limiting, and as such HLA-A2 transgenic mice are accurate predictors of human CTL responses [15], [17], [39], [43]. These studies were carried out in human HLA-A2 transgenic mice of two strains, with concordant results in both. Thus, if mouse CTL specific for these POTE peptides and HLA-A2 kill human tumor cells despite other differences in adhesion and accessory molecules, it is likely that human CTL specific for the same peptides and HLA-A2 will do so as well. In consideration of choosing the best target for immunotherapy, a combination of POTE 553-1Y and POTE 323-3F epitopes may represent an attractive vaccine strategy to avoid developing immune-escape variants as well as to overcome tolerance to wild type sequences.

In this study, we identified potential cancer vaccine targets in the POTE protein and demonstrated that CD8^+^ CTLs induced by POTE 553-1Y and POTE 323-3F can lyse human cancer cells expressing POTE. Future clinical trials are needed to verify the applicability of this approach in cancer patients.
